# Music to One’s Ears: Familiarity and Music Engagement in People With Parkinson’s Disease

**DOI:** 10.3389/fnins.2019.00661

**Published:** 2019-06-25

**Authors:** Ilene Berger Morris, Erin Vasudevan, Margaret Schedel, Daniel Weymouth, Jay Loomis, Tzvia Pinkhasov, Lisa M. Muratori

**Affiliations:** ^1^St. Charles Hospital, Port Jefferson, NY, United States; ^2^School of Health Technology and Management, Stony Brook University, Stony Brook, NY, United States; ^3^Consortium for Digital Arts, Culture, and Technology, Stony Brook University, Stony Brook, NY, United States; ^4^Department of Biomedical Informatics, Stony Brook University, New York, NY, United States; ^5^Department of Music, Stony Brook University, Stony Brook, NY, United States; ^6^School of Medicine, Stony Brook University, Stony Brook, NY, United States

**Keywords:** neuromusic, Brief Music Experience Questionnaire, neurologic music therapy, Rhythmic Auditory Stimulation, Parkinson’s disease therapy

## Abstract

Parkinson’s disease (PD) is a complex diagnosis commonly associated with motor dysfunction, but known to comprise cognitive, psychiatric, and mood disturbances as well. Music has been successfully used to address motor and non-motor symptoms of PD. Still, little is known about the nature of an individual with PD’s experience and relationship with music on conceptual and emotional levels, which may factor into their engagement in music-based techniques to ameliorate impairments. Two surveys were administered to 19 individuals with PD and 15 individuals without PD in order to gauge their subjective impressions and valuations of music. Participants completed The Brief Music Experience Questionnaire (BMEQ), a standard self-report measure pertaining to the role of music in one’s life, prior to performing a perception task which involved listening to and making sound adjustments to three music recordings. Following the perception task, a custom Exit Survey was administered to evaluate the experience of listening to and engaging with the music in the perception task. In all six dimensions of the BMEQ, examining aspects of music experience including commitment to music, self-reported musical aptitude, social uplift, affective reactions, positive psychotropic effects, and reactive musical behavior (RMB, pertaining to actions or behaviors in response to music), the mean and the median were greater for the control group than for the PD group, but the difference was only statistically significant in the RMB dimension. On the Exit Survey, both groups assessed recent, specific, and interactive music listening more positively than the imagined, hypothetical or general music experiences addressed on the BMEQ. Additionally, familiarity had a greater effect on listening pleasure for participants with PD than those without PD. We conclude that people with PD may perceive less of an automatic connection between music and activity than their healthy peers. Additionally, they may receive more pleasure and value from music than they anticipate. Taken together, our results suggest that people with PD may require encouragement to participate as well as empowerment to choose familiar selections in order to better access music-based interventions and the benefits they can offer.

## Introduction

Parkinson’s disease (PD) is characterized by degeneration of dopaminergic neurons and reduced innervation of the substantia nigra and the basal ganglia, neural structures that are responsible for generating the internal rhythm required for executing walking movements (Merchant et al., 2013; Dalla Bella et al., 2015). Gait disorders such as start hesitation, freezing of gait, and festination (small, rapid steps), along with postural instability, severely decrease independence and increase the risk of falls in PD (Clair et al., 2011; Grabli et al., 2012). Bradykinesia (slowness) and muscle rigidity, prominent motor symptoms of PD, also affect the muscles of the vocal apparatus, leading to speech dysfunctions such as slowness, breathiness, harshness, and limitations in pitch and loudness (Holmes et al., 2000; Pinto et al., 2004). PD can also impair cognition and lead to emotional disturbances such as depression and anhedonia, a lowered ability to experience pleasure (Loas et al., 2012).

Music has long been a part of the landscape in the treatment of PD. Applications of music and its elements have addressed the wide-ranging symptoms and functional deficits caused by PD, and can complement or exceed benefits achieved through other forms of treatment (Pacchetti et al., 2000; Nombela et al., 2013; Dalla Bella et al., 2015; Rodger and Craig, 2016). Music is processed diffusely throughout the brain, where networks for the processing of music and its components such as melody, pattern, meter, and tempo overlap with networks that govern other human functions (Thaut, 2008). For example, neural activity in rhythm perception is closely related to that of movement regulation, involving cortical and subcortical regions such as the premotor cortex, supplementary motor area, cerebellum, and the basal ganglia (Zatorre et al., 2007; Raglio, 2015). As many of these same areas are compromised in PD (Nombela et al., 2013), the ability of music to activate key motor regions during rhythm perception can serve an important compensatory purpose. Music can increase regional cerebral blood flow (Blood and Zatorre, 2001) and stimulate the release of dopamine (Salimpoor et al., 2011), a neurochemical depleted in PD, which also regulates motivation and goal-directed behaviors (Chanda and Levitin, 2013).

Internal cueing of movement timing is disturbed by malfunctioning basal ganglia – cortical circuitry in people with PD (Herrojo Ruiz et al., 2014). However, external auditory cuing in the form of metronome pulses or rhythmic music can enable affected individuals to initiate steps and maintain gait movements (Benoit et al., 2014; Mainka, 2015) or can train sequences of action related to everyday tasks (Pohl et al., 2013). In music with a clear beat, the steady temporal input serves as a continuous reference, creating a rhythmic template that influences the motor system’s ability to coordinate and execute movement (Nombela et al., 2013). As the pattern of regular external cues generates temporal expectations, the temporal-motor system begins to act on those expectations, predicting subsequent beats and priming movement in anticipation of them (Nombela et al., 2013; Benoit et al., 2014). In the absence of a healthy basal ganglia timing system, the cerebellar–thalamic–cortical network seems to be recruited to mediate the entrainment process, or synchronization of movement to sound (Thaut, 2008; Nombela et al., 2013; Benoit et al., 2014; Raglio, 2015). In addition, cueing through a neurologic music therapy technique known as Rhythmic Auditory Stimulation (RAS) has been shown to help normalize multiple gait parameters including velocity, cadence, and stride length (Thaut et al., 1996; McIntosh et al., 1997; Arias and Cudeiro, 2010) even on individuals with mild cognitive impairment (Rochester et al., 2009).

Research has shown that music that is familiar is more likely than unfamiliar music to lead to accurate tempo matching and functional strides with RAS (Leow et al., 2015). Familiarity influences emotional arousal; this level of arousal is strongly related to the degree of pleasure experienced by the music listener (van den Bosch et al., 2013). Predictions and expectations of auditory events are made and satisfied when listening to familiar music, resulting in dopamine release in the striatal system (Salimpoor et al., 2011), as well as activation of emotion-related regions (Pereira et al., 2011). Familiar, preferred music can optimize motivation for therapeutic training programs and promote emotional engagement (Mainka, 2015). For example, music therapy has been shown to mitigate speech impairment in PD by facilitating synchronization of articulatory muscle patterns to rhythm, and training respiratory support and control through singing and other vocal exercises. These types of programs are often provided in a group singing or choir context, allowing for rewarding social interaction and improving quality of life (Yinger and LaPointe, 2012; Buetow et al., 2014; Stegemöller et al., 2016). Cognitive abilities have also been improved through music-based training in this population (Pohl et al., 2013).

In addition to experiencing physical challenges, depression is common in people with PD (Frisina et al., 2008; Reijnders et al., 2008), and mood disorders may manifest even before motor symptoms appear (Raglio, 2015). Depression is considered a core symptom of PD, diminishing quality of life (Global Parkinson’s Disease Survey Steering Committee, 2002). Some researchers believe depression in PD is mediated by the degeneration of the neurotransmission of dopamine, among other neurochemicals (Sawabini and Watts, 2004). The ability of music to induce neurochemical and physiological changes may have particular relevance in the treatment of people with PD, not only for its effects on movement, but also to address mood disturbances (Koelsch, 2010; Bega et al., 2014). Enjoyable music recruits the reward-motivational circuitry involved in survival behaviors, with activations in areas including the ventral striatum and its nucleus accumbens (Blood and Zatorre, 2001). Music listening has been shown to trigger the release of dopamine in the striatal system (Salimpoor et al., 2011). Therefore, dopaminergic activity may be mediating the affective response to music through mesolimbic structures relevant to PD.

Earlier research shows that people with PD and their healthy counterparts are equally able to detect out-of-key tones, rhythmic changes, and differences in meter (Lima et al., 2013). In addition, through experimentation we have shown that individuals with PD are able to perceive and correct distortions introduced into different musical pieces, although they are slightly less able to eliminate distortions than healthy peers (Muratori et al., 2015; Pinkhasov et al., 2015; Schedel et al., 2016). In the current study, we were interested in seeing if individuals with PD experience music in the same way as those without PD. To do this we analyzed survey responses to a broad range of statements about personal music experiences and reactions to music in general and specific to listening to three musical pieces. We hypothesized the two populations would have equal familiarity with musical selections. However, due to the frequency of depressive symptoms in people with PD, we expected that participants with PD would return more negative survey ratings than those without PD, with specific differences in responses to involvement in musical behaviors and enjoyment resulting from listening to music.

## Materials and Methods

Nineteen individuals with PD (11 male, aged 52–79 years, $\bar{x}$ = 67 years, Hoehn and Yahr I–III) and fifteen healthy peers (7 male, aged 51–89 years, $\bar{x}$ = 66 years) participated in this study. Participants were recruited by word of mouth and via flyers with information sent to local movement disorder neurologists. Interested persons called the principal investigator (LM) and were screened for eligibility as either a person with PD or a peer without PD. Eligibility included (1) adequate visual and auditory acuity and motor control to perform the study tasks; (2) willingness and ability to sign an informed consent and comply with the study protocol; and for participants with PD; (3) clinical PD as determined by a neurologist; any disease duration and disease level according to the Hoehn and Yahr scale (Hoehn and Yahr, 1967); (4) no history of a secondary neurological or medical problem that has a known effect on vision, auditory, or cognitive functioning; and (5) stable neurological function and medications for at least 30 days prior to study entry. This study was carried out in accordance with the Committee on Research Involving Human Subjects, Institutional Review Board (IRB) of Stony Brook University. All participants gave written informed consent in accordance with the Declaration of Helsinki. The protocol was approved by the IRB prior to initiating any data collection. Testing occurred at Stony Brook University’s Rehabilitation Research and Movement Performance (RRAMP) Laboratory.

Upon arrival at the lab, participants were surveyed regarding their musical experience through a computerized version of the Brief Music Experience Questionnaire (BMEQ) (Werner et al., 2006) using Qualtrics software (Qualtrics, Provo, UT, United States). Completion of the BMEQ was immediately followed by listening to excerpts of three pieces of music representing a range of genres and textures: Billie Holiday’s “Love me or leave me” (Donaldson and Kahn, 1928/1941/1996, track 14), The Beatles’ “Here comes the sun” (Harrison, 1969, track 7), and Haydn’s “Finale – Allegro con spiritu” (Haydn, 1795/2008, track 8) from the 103rd Symphony. The Beatles song is in the genre of rock and roll. Its homophonic setting features prominent male vocals and acoustic guitar lines, with other components of a rock band providing harmonic support, and a tempo of 129 beats per minute (bpm). Haydn’s work is an example of classical instrumental music in a densely layered orchestral setting, at 145 bpm. The Billie Holiday song is from the jazz genre, with an instrumental ensemble backing up an expressive female vocal line. It has the slowest tempo of the pieces, at 90 bpm. We wanted to give participants a variety of musical affects to draw from, and provide different auditory backdrops against which the distortions would be detected. Each song was first played as it was originally recorded. Then (as part of a separate study – see Muratori et al., 2015; Schedel et al., 2016) three different kinds of distortions were overlaid onto each recording with Ableton Live Software (Ableton, Berlin) to create nine different musical conditions. Distortions consisted of Beat Repeat (a captured sound repeated in a loop for a jittery, shuddering effect); Timbral Shift (a high-frequency whooshing or warbling sound produced by a frequency shifter); and White Noise Generator (static). Each condition was repeated three times for a total of 27 trials. For more detail about the distortions, please refer to our prior publications (Muratori et al., 2015; Schedel et al., 2016). The procedure consisted of approximately 20 min of listening time per song; the song was distorted for about 5 min of this time. Rather than passively listening to music, the participants were required to actively listen to, interact with, and evaluate the effects of their responses on the music, resulting in a profound exposure to each musical selection. Immediately after the active listening, participants completed a pencil-and-paper Exit Survey, a custom measurement tool created by the researchers for purposes of this study, rating their familiarity with and enjoyment of the music heard. The results of the BMEQ and Exit Survey are reported here with a discussion of considerations for the use of music as an intervention in PD.

### Measures

The Music Experience Questionnaire (MEQ) was developed to measure the relationship between music experience and aspects of personality, including clinically relevant behavior (Werner et al., 2006, 2009). The developers of this self-report measurement tool suggested that such a questionnaire may be useful in the clinical setting to identify individuals who are likely to respond to music-based intervention techniques, and recommended that future MEQ-related studies factor in music preference and listening choices (Werner et al., 2009). Respondents’ choices on a five-point rating system represent their level of agreement with 141 survey statements, from 1 (Very untrue) to 5 (Very true). Statements on a range of music experience topics are intended to be relevant to non-musicians as well as musicians, and are grouped into six specific categories (also referred to as scales or dimensions) of music experience outlined by Werner et al. (2009) that focus on types of responses to and involvement in music: “Commitment to Music” (CM), “Social Uplift” (SU), “Affective Reactions” (AR), “Reactive Musical Behavior” (RMB), “Innovative Musical Aptitude” (IMA), and “Positive Psychotropic Effects” (PPE), as shown in Table 1. We used a condensed version of the MEQ, the BMEQ, which comprises 53 items falling within these categories.

**TABLE 1:** Brief Music Experience Questionnaire (BMEQ) dimensions.

**Abbreviations**	**Dimension**	**Description**
AR	Affective Reactions	Affective and spiritual reactions to music
CM	Commitment to Music	The centrality of pursuit of musical experiences in the person’s life
IMA	Innovative Musical Aptitude	Self-reports of musical performance ability as well as the ability to generate musical themes and works
PPE	Positive Psychotropic Effects	Calming, energizing, integrating reactions
RMB	Reactive Musical Behavior	Motile reactions including humming and swaying along with music
SU	Social Uplift	The experience of being stirred and uplifted in a group-oriented manner by music

*Adapted from Werner et al. (2006).*

Our Exit Survey listed the music played by title and performer or composer, to aid in identification and recall. The headings “The Beatles (Here Comes the Sun),” “Billie Holiday (Love Me or Leave Me),” and “Haydn (Symphony)” were followed by rows for indicating familiarity and enjoyment. As the BMEQ utilized a five-point rating system, we used the same system to express ratings of these variables. Guidelines were printed under the numbers to illustrate the direction of response strength. The familiarity scale ranged from “Never heard it before” to “Very familiar,” and the esthetic response scale ranged from “I hate it” to “I love it.” The participants circled the number, from 1 to 5, that represented their level of familiarity with and listening enjoyment of each of the three musical selections. The participants had the option to re-listen to any of the songs to ensure that they were attributing their reactions to the correct piece. Participants offered their ratings at the conclusion of the entire set of listening trials.

### Data Analysis

Descriptive statistics were computed for all variables and used for further analysis. Data was analyzed using Statistical Package for the Social Sciences software (SPSS, version 20, IBM, NY, United States). For the BMEQ, reverse-coded question scores were re-ordered so that all questions could be evaluated using a scale with a consistent direction. To determine differences between participants with and without PD, BMEQ and Exit Survey data was examined using Mann–Whitney *U* tests with a *p* < 0.05 significance level. The non-parametric Mann–Whitney test was chosen because response datasets did not, in general, conform to a normal data distribution. Mean responses for each participant were used as *U* test input in order to focus on the variance between subjects. BMEQ data was further divided to examine the influence of the six dimensions within the survey. As previous literature has shown an influence of familiarity on music enjoyment (Meyer, 1994; Pereira et al., 2011; van den Bosch et al., 2013), the Exit Survey data was analyzed using Spearman’s rho correlation testing to determine if participants with PD and control participants were equally impacted by the songs tested.

## Results

Data analysis revealed minor variation between populations, and one instance of statistical significance when examining responses at the level of each of the six scales that make up the BMEQ, though on this survey as a whole there was no response difference between populations (*p* > 0.05 across categories). These scales categorize reactions to and experiences of music that underlie the perceived role of music in one’s life, as outlined in Table 1.

**FIGURE 1:**
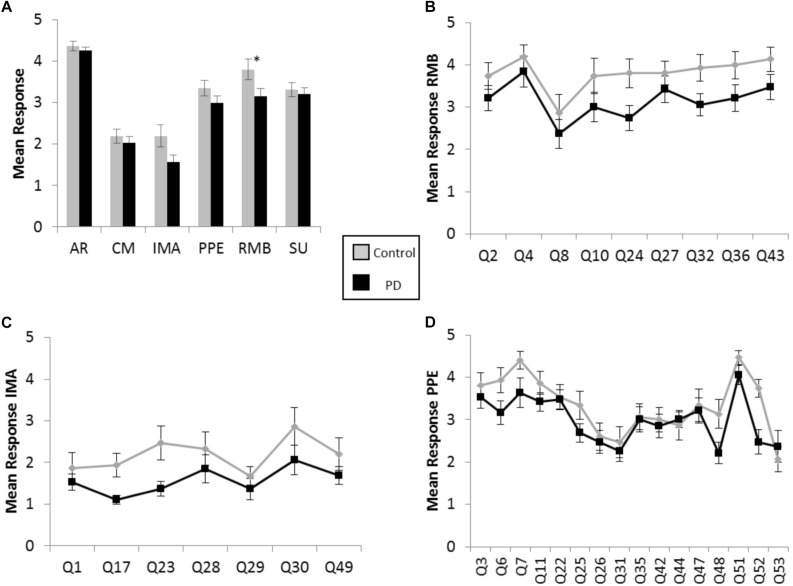
Brief Music Experience Questionnaire (BMEQ) ratings by participants with and without PD. **(A)** Overall group means of six music experience scales of the BMEQ showing the categories: Affective Reactions (AR), Commitment to Music (CM), Innovative Musical Aptitude (IMA), Positive Psychotropic Effects (PPE), Reactive Musical Behavior (RMB), and Social Uplift (SU) as rated on a 5-point scale (*y* axis) by participants with (Black) and without (Gray) PD. Error bars indicate the standard error of the means. Asterisks (^*^) indicate significance between groups at *p* < 0.05 as determined by Mann–Whitney *U* test. Individual question means for **(B)** RMB, **(C)** IMA, and **(D)** PPE for participants with and without PD. Q numbers (*x* axis) refer to the questions/items on the BMEQ that constitute the corresponding category. Data points represent mean responses for each participant group to that question.

There were greater positive responses by control participants to 43 out of 53 survey statements, or 81% of the questionnaire, with responses from the participants with PD lagging behind on each of the six scales, throughout the various contexts of music experience explored in the BMEQ (Figure 1A). Yet, a significant difference between groups was found in only the RMB dimension, in which individuals with PD reported lower responses than their non-PD peers (RMB PD median = 3.0, Control median = 4.1, *U* = 80, *Z* = −2.17, *p* < 0.05, *r* = 0.37). Note that these differences were seen throughout the RMB dimension questions rather than just resulting from a single divergent response (see Figure 1B). Figures 1C,D show that mean responses for the PD group also trailed the control group for questions throughout the IMA and PPE scales, though the difference in median response did not prove to be statistically significant in the Mann–Whitney *U* test (see Table 2).

**TABLE 2:** Two sample Mann–Whitney *U* test results for the domains of the BMEQ.

**Scale**	**PD median**	**Control median**	***U* statistic**	***z* score**	***p* value**	**Effect size**
AR Affective Reactions	4.30	4.40	118.0	−0.86	0.41	0.15
PPE Positive Psychotropic Effects	3.19	3.44	104.5	−1.32	0.19	0.23
CM Commitment to Music	1.86	2.29	117.5	−0.87	0.391	0.15
IMA Innovative Musical Aptitude	1.29	2.14	92.5	−1.75	0.083	0.30
RMB Reactive Musical Behavior	3.00	4.11	80.0	−2.17	0.030^*^	0.37
SU Social Uplift	3.25	3.50	129.0	−0.47	0.656	0.08

Asterisk (^*^) indicates statistically significant difference between groups at *p* < 0.05. For each participant, mean responses were calculated over all questions in the domain and used as input to the analysis (*N* = 34). Effect size *r* = *Z*/sqrt(*N*).

**FIGURE 2:**
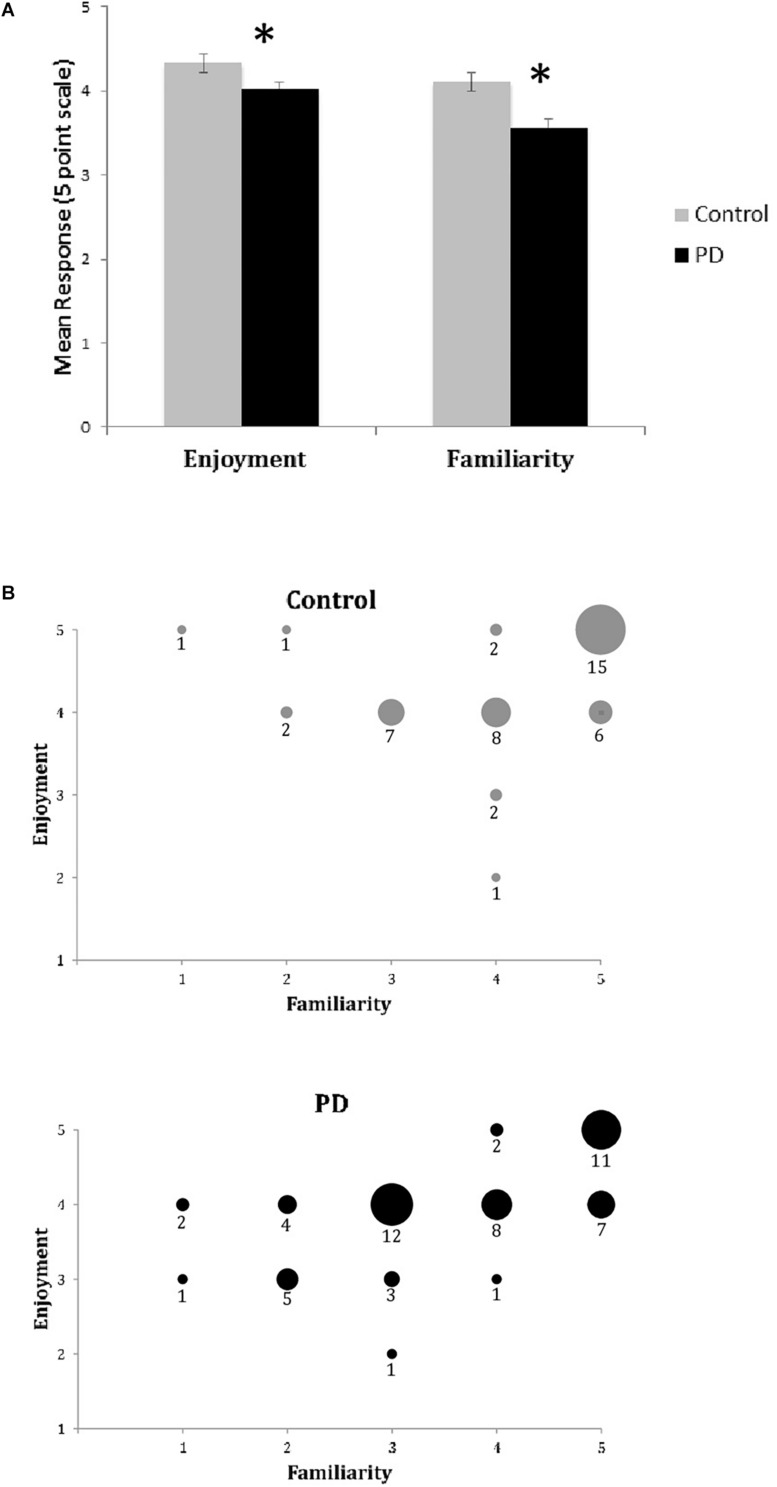
Exit Survey results of music enjoyment and familiarity. **(A)** Mean responses (±SEM) for enjoyment and familiarity on the Exit Survey for healthy controls (Gray) and participants with PD (Black). Asterisks (^*^) indicates significance at *p* < 0.05 as determined by Mann–Whitney *U* test. There were significant between group differences for both enjoyment and familiarity. **(B)** Enjoyment plotted against familiarity for control (top) and PD (bottom) groups. Responses for all songs and all participants are included in dataset. Size of circle and numbers below indicate the number of participants from each group who responded at that coordinate for the three songs.

Unlike the BMEQ, the custom Exit Survey evaluated responses as they related to the specific recent experience of listening to the three musical pieces in this experiment through ratings on two parameters: familiarity and enjoyment. Most participants reported familiarity with and enjoyment of the selected pieces with overall $\bar{x}$ scores of 3.8 and 4.2 on the respective 5 point scales. However, similar to the BMEQ, the Exit Survey showed a moderate effect size with results that were lower for participants with PD, indicating those with PD were less familiar (PD median = 3.67, Control median = 4.33, *U* = 78, *Z* = −2.28, *p* < 0.05, *r* = 0.39) and enjoyed the music less (PD median = 4.0, Control median = 4.3, *U* = 85, *Z* = −2.10, *p* < 0.05, *r* = 0.36) than their healthy peers (see Table 3). Examining the influence of familiarity on enjoyment, bivariate correlation analysis demonstrated an overall correlation between familiarity and enjoyment (Spearman’s rho = .581, *p* < 0.001) with a stronger effect for those with PD (Spearman’s rho = 0.654, *p* < 0.001) than for control participants (Spearman’s rho = 0.458, *p* = 0.002; see Figures 2A,B).

**TABLE 3:** Two sample Mann–Whitney *U* test results for the Exit Survey.

**Scale**	**PD median**	**Control median**	***U* statistic**	***z* score**	***p* value**	**Effect size**
Familiarity	3.67	4.333	77.5	−2.28	0.023^*^	0.39
Enjoyment	4.00	4.333	84.5	−2.10	0.043^*^	0.36

Asterisk (^*^) indicates statistically significant difference between groups at *p* < 0.05. For each participant, mean responses over the three Exit Survey songs were used as input to the analysis (*N* = 34). Effect size *r* = *Z*/sqrt(*N*).

## Discussion

Differences in responses on the BMEQ between the two populations surveyed were subtle but consistent. On the majority of questions, ratings from participants with PD were lower than controls on a broad range of items pertaining to musical responsiveness, consciousness, ability, and overall experience. Responses from healthy participants were skewed toward the high end of the five-point scale denoting more positive responses to or agreement with the statements, whereas responses from those with PD veered more toward the low end, even if just slightly in most dimensions. However, only in items dealing with motor/behavior responses to music, represented in the RMB category of BMEQ survey questions, was the difference between groups significant. PD causes disturbances in the motor and vocal systems that support these types of musical responses. Whereas healthy people may feel stimulated or even compelled to sing or dance along to music under certain circumstances, members of our PD group reported they saw little influence on such activities from music. The generation of motor responses related to anticipation of reward appears to be mediated by the release in the ventral striatum of dopamine, which is depleted in PD (Elliott et al., 2004). When diminished dopaminergic activity interrupts the normal circuit of anticipation, movement and reward in PD, individuals may not feel moved to move. Our data suggests that people with PD perceive the motor impairments caused by the disease as pervading their physical responses to music.

Depression and anhedonia, more common in PD than the broader population (Frisina et al., 2008; Loas et al., 2012), may translate to a reduction in behaviors and activities that seek, explore, express, and result in pleasure. Although we did not evaluate participants for these conditions, it is possible that depression in some participants with PD resulted in lower scores.

Meaningfulness, esthetics, emotional response to and valence of music are covered in the BMEQ’s AR scale, which returned the smallest average difference between groups and had the most positive responses of all the categories. AR casts music as a general concept as well as a stimulus capable of evoking subjective, affective/spiritual reactions. In AR, the PD group’s impressions of music’s power to mediate responses in the emotional realm may be seen as suppressed compared to controls, but to a noticeably lesser degree than on the scale dealing with physical/behavioral responses and capacities (RMB), or even creative/expressive and calming/energizing effects (IMA and PPE, respectively). This is somewhat surprising as AR has been correlated with the Center for Epidemiological Studies Depression Scale in previous studies (see Werner et al., 2009) and a stronger distinction between groups was expected in our hypothesis based on an increased likelihood of depression in the PD group. Although it is certainly possible that our sample did not exhibit depression overall, an alternate suggestion is that for people with PD, the ability to relate and respond to music on an emotional level may exceed their estimation of its capability to influence other aspects of their lives.

Our Exit Survey was concerned solely with listening data, and in particular with familiarity and enjoyment ratings of discrete works listened to in the immediate past (the Exit Survey was completed just after the listening trials). Importantly, all participants had repeated exposure to each of the three songs as part of the active listening trials. In a study by Madison and Schiölde (2017), novel musical examples that were initially less liked by listeners increased in enjoyment ratings after multiple presentations, demonstrating increased enjoyment with increased familiarity. In our study, control participants reported higher pleasure rankings on the Exit Survey than participants with PD. We did not explore whether the distortions, which were added to the original recordings for brief periods as part of a separate study (Muratori et al., 2015; Schedel et al., 2016), may have affected pleasure or familiarity valuations by either or both groups of participants. However, it is possible that the distorted sounds had different effects on each group and perhaps biased the findings. Uluyol et al. (2016) reported that individuals with PD have more high frequency hearing loss and greater severity of tinnitus than peers without PD. In addition, central auditory processing deficits in PD may have influenced perceptions of distortions making them less impactful in our participants with PD (Folmer et al., 2017). As those with PD may not have heard the full range of distorted sounds or may have processed the distortions less completely, reports of more enjoyable experiences on the Exit Survey may reflect this difference.

The three pieces of music were chosen in advance by the researchers without knowing what preferences and dislikes or emotional associations with the music the participants might have had. The use of existing, available music gave us the ability to consider the effects of familiarity, but also brought with it the possible influence of episodic musical associations (Sloboda, 1999). The experience of hearing certain music connects the listener with previous events that the person associates with the music, and the people, places, and emotions that played a part in them. These memories may be extramusical, distinctly individualized, and highly charged, emotionally. Therefore, the specific selections may have affected the listening experiences in ways that we were not able to predict ahead of time or evaluate in the data.

While we had hypothesized all participants would have similar previous exposure to the musical selections, those without PD also had unexpected higher rates of familiarity with the musical selections, with greater listening enjoyment during the task. Not surprisingly, the Beatles selection was well known to all the participants, but the Haydn and Billie Holiday pieces were both more familiar to the control participants than to those with PD. While the overall relationship between familiarity and enjoyment was not surprising given previous studies (e.g., van den Bosch et al., 2013) the strength of this relationship was greater for the participants with PD. Listening proximity may have been a factor that prompted a different and more positive interpretation and valuation of the listening experience, as both respondent groups reported higher levels of emotional engagement in the immediate post-experimental survey (Exit Survey) compared to the pre-test survey of hypothetical or imagined music listening and consumption (BMEQ). Immediacy and familiarity may be necessary for people with PD to cross the threshold from indifference to arousal, excitement and uplift from music, which may mean that they will be more likely to seek out and benefit from the therapeutic effects of applications of music.

Results demonstrated that following a recent specific music listening exercise, music was viewed more positively (measured on the Exit Survey) compared to general experiences of music on the earlier-administered BMEQ. We believe that the task of actively engaging with the music in listening trials versus abstractly thinking about music may explain this difference. Unlike passive listening (such as hearing background music), active listening involves listening *for* as much as listening *to* the music. The active listener mentally tracks the music through time, maintaining attention and engaging cognitive processes beyond the simple perception of sound (Gregory, 2002). Attentive music listening is linked with psychophysiological arousal, and a strong positive correlation exists between arousal and pleasure ratings (Salimpoor et al., 2009). Particularly relevant to people with PD, this arousal effect can influence motivational processing, and, indeed, therapeutic applications of music have been shown to increase motivation in people with poor ability to internally generate feelings of anticipation, motivation, and drive (Pacchetti et al., 2000). In addition, it is possible that the motor aspect of the listening task (moving controls on an iPad) contributed to all participants attending to the music with more intensity that would have been achieved without the movement element. A sustained active music listening intervention with movement features has been shown to help maintain attention skills for people with diagnoses, like PD, that affect cognitive processing (Gregory, 2002). In the movement task associated with our study, manipulating the slider and adjusting the sound may have mimicked the experience of playing an instrument, a motor-attentional operation that combines stimulation of both auditory and tactile pathways for a more integrated sensory response (Pacchetti et al., 2000).

We saw that familiar music in particular can catalyze a pleasure response in people with PD, consistent with findings in the general population by Pereira et al. (2011) and van den Bosch et al. (2013). Music responses that operate below the conscious level may have been at play, causing a distinction between contextual music experiences and imagined music listening, with additional valence generated by familiarity. In therapeutic applications, music can stimulate both conscious and automatic processes to alleviate symptoms and improve quality of life (Pacchetti et al., 2000; Clair et al., 2011). Some reactions to music occur without conscious awareness or intent, such as RAS entrainment effects, arousal and motivational benefits and physiological changes. As we have seen, even though the qualities of certain aspects of music, such as its ability to elicit an affective response, may be viewed quite similarly by individuals with PD and their unafflicted peers, music listening experiences vary with personal perceptions. This has implications for clinical work when music is used in interventions with persons with PD. Familiarity and preference of music should be considered. Furthermore, while survey assessments or other quantitative evaluation tools may be useful in identifying candidates for music therapy and the kinds of music to which they would most favorably respond (Werner et al., 2009), it is important to note the limitations of these tools in capturing subjective, experiential information. Future studies incorporating qualitative methods could greatly enhance our understanding of how people with and without PD experience and appreciate music.

One limitation of the present study is that the investigators were not blinded as to whether the participant had PD. Note that the surveys were completed by the participant on a computer (BMEQ) or with pencil-and-paper (Exit survey), and there was limited interaction with the investigators during the completion of these surveys. Furthermore, the quantitative nature of these surveys leaves little room for subjective interpretation by the investigators during analysis. We cannot, however, rule out the possibility that the investigators could have unintentionally influenced the results. The effect of participant-investigator interaction on the musical experience would be an interesting topic for future research, and may provide useful insights into the effectiveness of music therapy. For instance, one might ask whether the clinician’s music preference affects the person’s response to music therapy.

Another limitation of the present study is that we examine how people with PD respond to only one type of musical experience: listening. Other avenues of research could investigate whether responses differ between different types of musical experience, such as dancing or music-making.

## Conclusion

Many elements factor into an individual’s musical preferences, enjoyment and consciousness; music enjoyment is a highly personal human reaction, based on an individualized framework shaped by emotions and subjective experience. In PD, there are disease processes that affect emotional state and enjoyment, and may influence an individual’s anticipation and expectations of music experiences. Past evidence has shown that people with PD can benefit from the rhythm and structure of music to train and enhance movement, speech abilities, cognitive function, and emotional well-being, and that familiarity may improve music therapy outcomes. Our data suggests that of the various parameters of music experience, the active, physical response is most keenly felt to be reduced in PD. It also appears that persons with PD may have a diminished perception of their ability to derive value or pleasure from music, but their capacity for enjoyment can exceed the expectation. As people with PD may underrate the value of music in their lives, they may need and benefit from encouragement to actively engage in music, to access its power to assist with movement and communication and to improve mood, motivation, and quality of life. Our analysis clearly suggests a particular dependence of enjoyment on music familiarity in PD, endorsing the use of client-preferred familiar music in music therapy applications for individuals with this disease.

## Ethics Statement

This study was carried out in accordance with the Committee on Research Involving Human Subjects, Institutional Review Board (IRB) of Stony Brook University with written informed consent from all subjects. All subjects gave written informed consent in accordance with the Declaration of Helsinki. The protocol was approved by the IRB prior to initiating any data collection.

## Author Contributions

All authors made substantial contributions to the conception, analysis, and interpretation of data of this work. IM, LM, MS, JL, and TP were involved in the acquisition of data. IM, MS, and LM provided the original drafts and all authors revised or provided the critical content to the paper prior to giving final approval of the manuscript. The authors agreed to be accountable for all aspects of the work in ensuring that questions related to the accuracy or integrity of any part of the work are appropriately investigated and resolved.

## Conflict of Interest Statement

The authors declare that the research was conducted in the absence of any commercial or financial relationships that could be construed as a potential conflict of interest.
